# Brain entropy and human intelligence: A resting-state fMRI study

**DOI:** 10.1371/journal.pone.0191582

**Published:** 2018-02-12

**Authors:** Glenn N. Saxe, Daniel Calderone, Leah J. Morales

**Affiliations:** Department of Child and Adolescent Psychiatry, New York University School of Medicine, New York, New York, United States of America; University of Cambridge, UNITED KINGDOM

## Abstract

Human intelligence comprises comprehension of and reasoning about an infinitely variable external environment. A brain capable of large variability in neural configurations, or states, will more easily understand and predict variable external events. Entropy measures the variety of configurations possible within a system, and recently the concept of brain entropy has been defined as the number of neural states a given brain can access. This study investigates the relationship between human intelligence and brain entropy, to determine whether neural variability as reflected in neuroimaging signals carries information about intellectual ability. We hypothesize that intelligence will be positively associated with entropy in a sample of 892 healthy adults, using resting-state fMRI. Intelligence is measured with the Shipley Vocabulary and WASI Matrix Reasoning tests. Brain entropy was positively associated with intelligence. This relation was most strongly observed in the prefrontal cortex, inferior temporal lobes, and cerebellum. This relationship between high brain entropy and high intelligence indicates an essential role for entropy in brain functioning. It demonstrates that access to variable neural states predicts complex behavioral performance, and specifically shows that entropy derived from neuroimaging signals at rest carries information about intellectual capacity. Future work in this area may elucidate the links between brain entropy in both resting and active states and various forms of intelligence. This insight has the potential to provide predictive information about adaptive behavior and to delineate the subdivisions and nature of intelligence based on entropic patterns.

## Introduction

This article describes a study on the relationship between human intelligence and fMRI brain entropy. There are several reasons to hypothesize that brain entropy would be related to intelligence. Entropy is a measure of the number of ways a system can be configured (i.e. the number of states to the system) [1]. An intelligent system models the information it receives through specific physical reconfigurations of its components in the form of system states [2–5]. The brain uses information by creating specific models of sensory input through specific readjustments of neuronal connections. These adjustments in neuronal connection are integrally related to learning, and the stability of these reconfigurations over time, for later access, are integrally related to memory [6–8].

Central to the concept of brain entropy is the concept of a brain state (or sub-state). It is clear that the brain uses stable configurations of neuronal connection to model and process information and to initiate adaptive behaviors in response to information received [8–11]. These processes are included in most definitions of brain state, even though there is no universally accepted definition for this concept. The concept of brain state will be clarified over time with research that elucidates such qualities as the specific density and distribution of neurons contained within a state, the patterns of their connectivity, the mechanisms by which they achieve stability over time, and the process by which they become engaged in response to specific demands. Notwithstanding the need to clarify these important aspects of a brain state, this concept has utility in defining important features of brain function, and the number of brain states that are accessible for brain functioning defines the concept of brain entropy. We use the working definition of brain state, and its role in brain functioning, proposed by Zagha and McCormick [11]: “We consider [brain] state to be a recurring set of neural conditions that is stable for a behaviorally significant period of time …”, fluctuations in multiple states and sub-states result in “…a highly dynamic and complex control of network responsiveness and processing in relation to behavior”. Zagha and McCormick conclude: “A major task for neuroscience is determining exactly how many sub-states exist and how they organize, interact and influence behavior” [11].

How might the concepts of brain state and brain entropy be related to human intelligence? Intelligence is generally thought to comprise the capacity “…to reason, plan, solve problems, think abstractly, comprehend complex ideas, learn quickly and learn from experience. …it reflects a … capability for …comprehending our surroundings—‘catching on,’ ‘making sense’ of things, or ‘figuring out’ what to do” [12]. Each of these capacities would require the creation of accurate predictive models of the world so that the brain is able to use the sensation it receives for adaptive action. Friston and Buzsaki [13] offer the term “Good-enough Brain” to describe the brains capacity to conduct Bayesian reasoning via the creation of accurate predictive models of the world through patterns of neuronal connectivity: “A key theoretical development in neurobiology is the appreciation of the brain as a predictive organ generating predictions of its actions and sensations. These predictions rest on an internal or generative model of how sensory input unfolds. One can understand much of neuronal dynamics and synaptic plasticity as an optimisation of (Bayesian) model evidence as scored by proxies like free energy and prediction errors. If one subscribes to this normative theory, the brain must be a good (enough) model of its environment, where recurring sequences of events are the rule” [13]. In order for a human brain to be “good-enough” to create accurate predictive models of the ‘recurring sequence of events’ it encounters, it would need to have access to a very large number of brain states that could be used to correlate with the great many recurring sequence of events it might encounter. This access should be integrally related to human intelligence. Thus, we hypothesize that intelligence is positively related to brain entropy.

A complete accounting of brain entropy would require knowledge of how brain architecture may allow the flexibility to create and store the very large number of distinct configurations that would be used to model and process the information the brain would receive over the life-course, and knowledge on how these configurations become accessed as brain states in appropriate contexts. There are important research advances that provide intriguing clues on these matters. For example, Chklovskii and colleagues [14] describe how neuronal connectivity, through weight and, especially, wiring flexibility can enable enormous capacity for storage of information. Lim and colleagues [15] demonstrated that storage and access to such stable neuronal connections are subject to very specific learning rules. Buzsaki has written extensively on how oscillatory processes are used to create temporal stability in the activity of specific groups of neurons for specific functional purposes [8, 9].

The technology to measure brain entropy has advanced over the last five years resulting in a growing literature using a variety of brain imaging techniques applied to understand normal and abnormal brain function [16–18]. Brain entropy can be measured from fMRI data, which consists of a series of successive MRI brain images taken over time. Each three-dimensional image is composed of individual voxels which contain a single value reflecting the blood-oxygen-level dependent (BOLD) signal of a small region. By examining a single voxel over the course of the fMRI scanning session, changes in the BOLD signal intensity of that small region can be tracked over time. Brain entropy is calculated as the predictability of single voxel signals over time. Although this procedure does not directly measure the number of specific brain states that are accessible during the measurement period, there is a very close relation between number of possible states to a system and the predictability of the behavior of the components (e.g. voxel signals) of a system. Predictability relates to the concept of information density described by Shannon [2]. In a less predictable time-series, with high entropy, each successive data point adds new information. In a low entropy time-series, the same information is repeated. Thus, a voxel whose BOLD signal intensity varies unpredictably over time would be given a high entropy value, whereas a voxel whose signal varies little, or in a highly regular pattern, would produce a low entropy value.

The data set used in this study includes resting-state fMRI data along with measures of intelligence. Using resting-state data to study intelligence makes sense from an entropic perspective, as we hypothesize that intelligence is driven by the brain’s capacity to access and deploy a large number of brain states to respond to unpredictable situations and challenges from the external environment. Measurement of brain entropy at rest would, in theory, provide a measure of the brain’s overall flexibility or readiness to encounter unpredictable stimuli. The brain, at rest, is still processing information in the form of thought, planning and reasoning: although it is not responding to specific environmental demands. Accordingly, measuring brain entropy at rest maybe more likely to capture the breadth of possible brain states than would measurement during a particular challenge/task when entropy is more likely to be narrowed considerably by task demands. On the other hand, entropy measured during a specific task performance may correlate more strongly with that task, since it captures the breadth of brain states actually utilized during performance. While the data set under investigation here does not include fMRI measured during active tasks, future work would benefit from examining brain entropy in both the resting state and during task engagement.

Two measures were used to estimate intelligence in this study: the Shipley-Hartford vocabulary task [19] and the matrix reasoning task from the Wechsler Abbreviated Scale of Intelligence (WASI) [20, 21]. IQ estimated from the Shipley task represents verbal intelligence, which relies on access to stored knowledge, a form of crystalized intelligence. Several brain areas, predominantly in the left hemisphere, have been linked to the semantic retrieval processes involved in vocabulary tasks [22–25]. The left middle and inferior temporal lobe, as well as the left angular gyrus, have been associated with semantic retrieval in language. The left inferior occipital and fusiform areas have been linked to orthographic information processing. The left middle frontal gyrus has been implicated in goal direction and cognitive control during word retrieval. In the resting state, we expect entropy in regions supporting semantic retrieval to indicate readiness to process vocabulary, rather than entropy in regions supporting cognitive control that might be more engaged by an active task. IQ estimated from the WASI matrix reasoning task represents performance intelligence, which relies on the flexible ability to solve novel problems. Such fluid intelligence has been related to activity in the bilateral superior, inferior, and middle frontal gyri, as well as anterior cingulate and paracingulate cortex [26, 27]. Visuospatial reasoning, specifically involved in the matrix reasoning task, is also linked to prefrontal cortical activity, as well as with activity in primary visual and parietal areas [28]. In the resting state, we expect entropy in the prefrontal cortex underlying fluid intelligence to predict readiness to engage in matrix reasoning, rather than entropy in visual and parietal areas that might be engaged when viewing specific stimuli.

## Materials and methods

### Experimental design

The analyses included data from 892 participants who had both fMRI resting-state scans and both types of IQ estimates. Data were provided by the Brain Genomics Superstruct Project (GSP) of Harvard University and the Massachusetts General Hospital, (Principal Investigators: Randy Buckner, Joshua Roffman, and Jordan Smoller), with support from the Center for Brain Science Neuroinformatics Research Group, the Athinoula A. Martinos Center for Biomedical Imaging, and the Center for Human Genetic Research. Twenty individual investigators at Harvard and MGH generously contributed data to the overall project. The data set contains resting-state fMRI scans and intelligence testing from 892 healthy adults between the ages of 18 and 35 (21.61 ± 2.84, mean ± standard deviation). Our analysis of this data set was approved by the NYU Langone Medical Center IRB. Written informed consent was obtained from subjects after the nature of the study and possible consequences were explained [29]. fMRI time-series data were preprocessed and summarized by calculating entropy for each voxel, creating a 3-dimensional brain image composed of entropy values. These images were used in regression analyses to determine whether brain entropy values predicted the IQ estimates.

### Intelligence measurement

Intelligence was measured with the vocabulary task from the Shipley Institute of Living Scale [19] and the Matrix Reasoning subtest of the Wechsler Abbreviated Scale of Intelligence (WASI) [20]. Shipley-Hartford Age-Corrected T-Scores from the vocabulary task were used to estimate full-scale IQ (FSIQ). Separately, matrix reasoning scores and demographic variables were also used to estimate FSIQ using the OPIE-3 formula, which has been shown to reliably predict FSIQ using matrix reasoning [30]. A subset of thirty-three GSP participants completed the full Wechsler Abbreviated Scale of Intelligence (WASI) and showed strong correlation between WASI FSIQ and the two estimated intelligence measures. Due to participant recruitment from Boston area universities and colleges, the average estimated FSIQ was elevated (110.7 ± 6.7) compared to general population estimates [30]. Average years of education (± standard deviation) was 14.59 ± 1.94. While both intelligence estimates sought to approximate FSIQ, Shipley estimates more directly assess verbal intelligence (vocabulary), and WASI estimates more directly assess performance intelligence (matrix reasoning). Average (± standard deviation) Shipley estimated IQ was 113.76 ± 8.81, and average WASI estimated IQ was 108.72 ± 8.15.

### MRI data parameters

MRI data acquisition and initial processing is described in detail in Holmes et al. [29]. All MRI data were obtained with 3T Tim Trio scanners (Siemens Healthcare, Erlangen, Germany) at Harvard University and Massachusetts General Hospital. MRI scans for each participant included a high resolution structural scan (T1-weighted multi-echo MPRAGE, TR = 2.2 sec, TE = 1.5/3.4/5.2/7.0 msec, slices = 144, resolution = 1.2 x 1.2 x 1.2 mm) and a resting-state functional scan sensitive to blood oxygenation level-dependent (BOLD) contrast (TR = 3.0 sec, TE = 30 msec, slices = 47, resolution = 3.0 x 3.0 x 3.0 mm, 120 measurements). Structural and functional scans included full coverage of the cerebrum and cerebellum.

### fMRI preprocessing

As part of the GSP project, functional scans were quality checked and screened for artifacts, acquisition problems, processing errors, and excessive motion. Scans that passed screening were motion corrected as described in Holmes et al. [29]. Functional scans were additionally processed for the current study using the AFNI [31] and FSL [32] software packages. Images were corrected for slice timing to the first slice, motion corrected to the middle image in the scanning sequence using a two-pass Fourier interpolation, deobliqued to a cardinal grid based on the minimum voxel spacing, despiked using the default settings in AFNI’s 3dDespike, and smoothed spatially (Gaussian blur, FWHM = 4.5 mm) using AFNI. Images were normalized using FSL (mean = 100, per each 3D volume).

### Sample entropy

Brain entropy was calculated using the Brain Entropy Mapping Toolbox (BENtbx) [33] for MATLAB (MATLAB Release R2015b, The MathWorks Inc., Natick, MA, United States). The BENtbx utilizes Sample Entropy (SampEn), an extension of Approximate Entropy, which is stable for data such as fMRI time-series. For a mathematically detailed description of the SampEn calculation, see [33].

For a given time-series, SampEn is a single number representing the predictability of the series. Given any particular value in a series, with what certainty can we predict its other values? In other words, how regular or irregular is the series? Is the series ordered or disordered? With a perfectly ordered series, such as [5, 5, 5, 5, 5,…], [5, 3, 5, 3, 5,…], or [5, 4, 1, 4, 5, 4, 1, 4, 5,…], given the value 5, the preceding and ensuing sequences are perfectly predictable. The entropy of highly predictable series is small, close to 0, indicating a lack of variation or disorder. With an unpredictable series, such as [3, 109, 7, 5–22,…], given the value 5, the rest of the series is difficult to predict. The entropy of unpredictable series is large, indicating a high amount of variation or disorder.

The Sample Entropy process first breaks a series into smaller sets of size *m*. For example, for *m* = 2, the BOLD time-series is broken into pairs of consecutive values. Each pair is then compared with every other pair to find the maximum distance (absolute value difference) between any number in the first pair and any number in the second pair. For example, for pairs [2, 5] and [4, 6], the maximum distance is between 2 in the first pair, and 6 in the second, and equals 4. If the distance is less than the threshold *r*, the two pairs are considered a ‘match.’ For example, for *r* = 3, [2, 1] and [3, 1] have a maximum distance of 2, and are thus a match. This process is then repeated for sets of size *m* + 1. Sample Entropy is then the ratio:

$$SampEn=-log\frac{A}{B}$$ (1)

where,

A=numberofmatchesusingsetsofsizem+1 (2)

B=numberofmatchesusingsetsofsizem (3)

For perfectly predictable series, *A* and *B* will be equal, and entropy will be 0. As disorder in a series increases, *B* will become greater than *A*, and the equation will yield an increasingly large positive number.

### Brain Entropy Mapping Toolbox analysis

Brain entropy was calculated using the Brain Entropy Mapping Toolbox (BENtbx) [33] for MATLAB (MATLAB Release R2015b, The MathWorks Inc., Natick, MA, United States). In the current study, *m* = 3, and *r* = 0.6 multiplied by the standard deviation of the data, which are optimal parameter values for resting-state fMRI data according to the BENtbx release paper [33]. Preprocessed resting-state fMRI images were analyzed with the BENtbx, which calculated the entropy of each time series within each voxel, producing a 3D image with an entropy value in each voxel. This image was then normalized using FSL and spatially smoothed using BENtbx (Gaussian blur, FWHM = 10).

### Statistical analysis

Brain entropy maps for 892 participants were entered into two separate whole-brain regression analyses using AFNI to test whether brain entropy was predicted by each of the two FSIQ estimates. The AFNI program 3dFWHMx was used to calculate the average autocorrelation function for the data, and this function was used by the program 3dClustSim to determine the minimum size of a voxel cluster needed for a corrected *p* of 0.001. Clusters were defined as groups of voxels above the uncorrected significance threshold whose faces (as opposed to edges or corners) touched. The results of the regression analyses were examined at an uncorrected threshold of *p* = 0.005 and a minimum cluster size of 157 voxels, resulting in a corrected *p* value of 0.001. The use of AFNI to accurately determine significance thresholds has recently been questioned. Eklund, Nichols, and Knutsson [34] showed that AFNI had a high family-wise error rate for a cluster defining threshold of *p* = 0.01, though performance was better for *p* = 0.001. Additionally, the authors noted that when a long-standing bug in 3dClustSim had been fixed, performance further improved. For these reasons, we used a stringent uncorrected voxel-wise threshold (*p* = 0.005), a new option in 3dClustSim to estimate the autocorrelation function of our data rather than relying on a Gaussian estimate, and a cluster defining threshold of *p* = 0.001.

Additionally, we repeated our regression analyses using SPM [35], an alternative software using parametric methods, with an uncorrected *p* = 0.001 and a cluster defining threshold of *p* = 0.001. We also performed the regressions with FSL’s “randomise” program, a nonparametric permutation test, with 500 permutations and threshold-free cluster enhancement (*p* = 0.005). The results of these analyses were compared to the results from AFNI. While clusters from these alternate analyses were similar to those derived from AFNI, the largest clusters were found with AFNI. Due to the exploratory nature of this study, we used the larger AFNI clusters for further analysis in which average cluster entropy was submitted to multiple regression.

## Results

**Fig 1:**
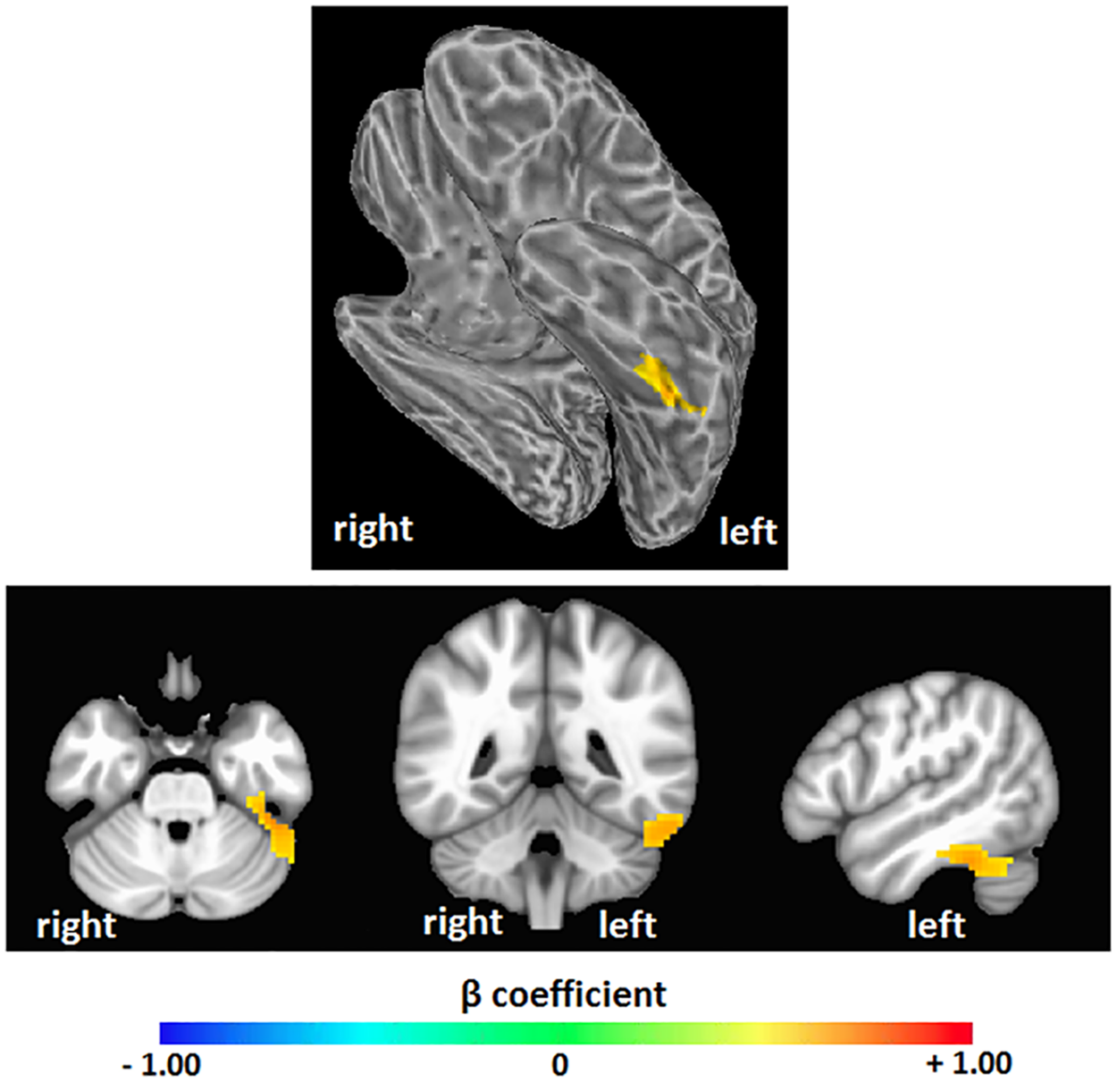
Brain entropy regression: Shipley estimated IQ. Whole brain regression analysis was performed with AFNI to determine whether brain entropy predicts full scale IQ as estimated from the Shipley Institutes of Living Scale, Vocabulary Task (uncorrected *p* = 0.005, cluster size = 157 voxels, corrected *p* = 0.001). Positive beta coefficients show where increases in resting-state brain entropy predict increases in IQ.

**Fig 2:**
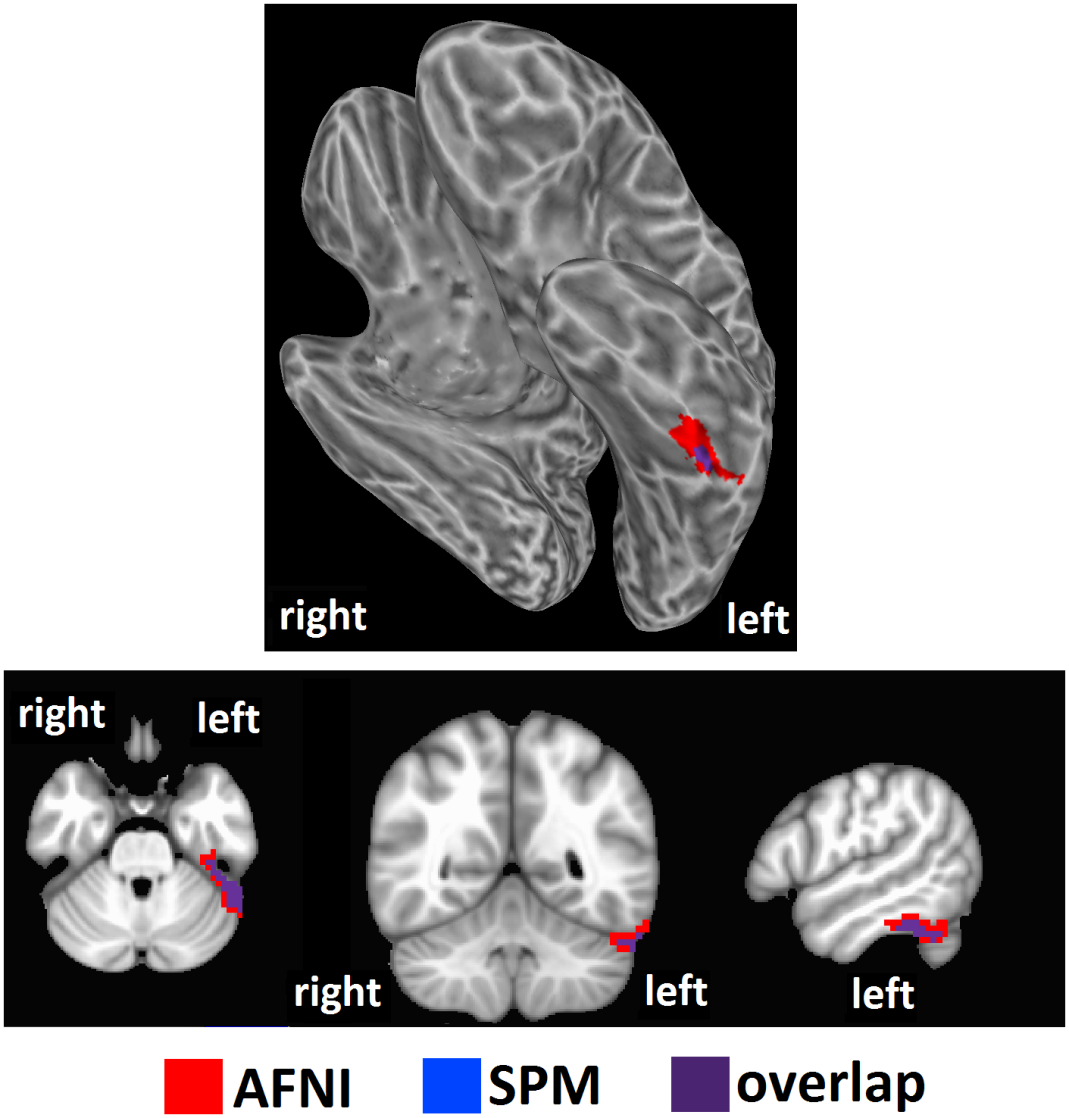
Brain entropy regression comparison with SPM: Shipley estimated IQ. Whole brain regression analysis with brain entropy predicting Shipley estimated IQ was performed with SPM to compare to the AFNI results (uncorrected *p* = 0.001, cluster size = 63 voxels, corrected *p* = 0.001). Results were in the positive direction, indicating increases in entropy predicting increases in IQ.

**Fig 3:**
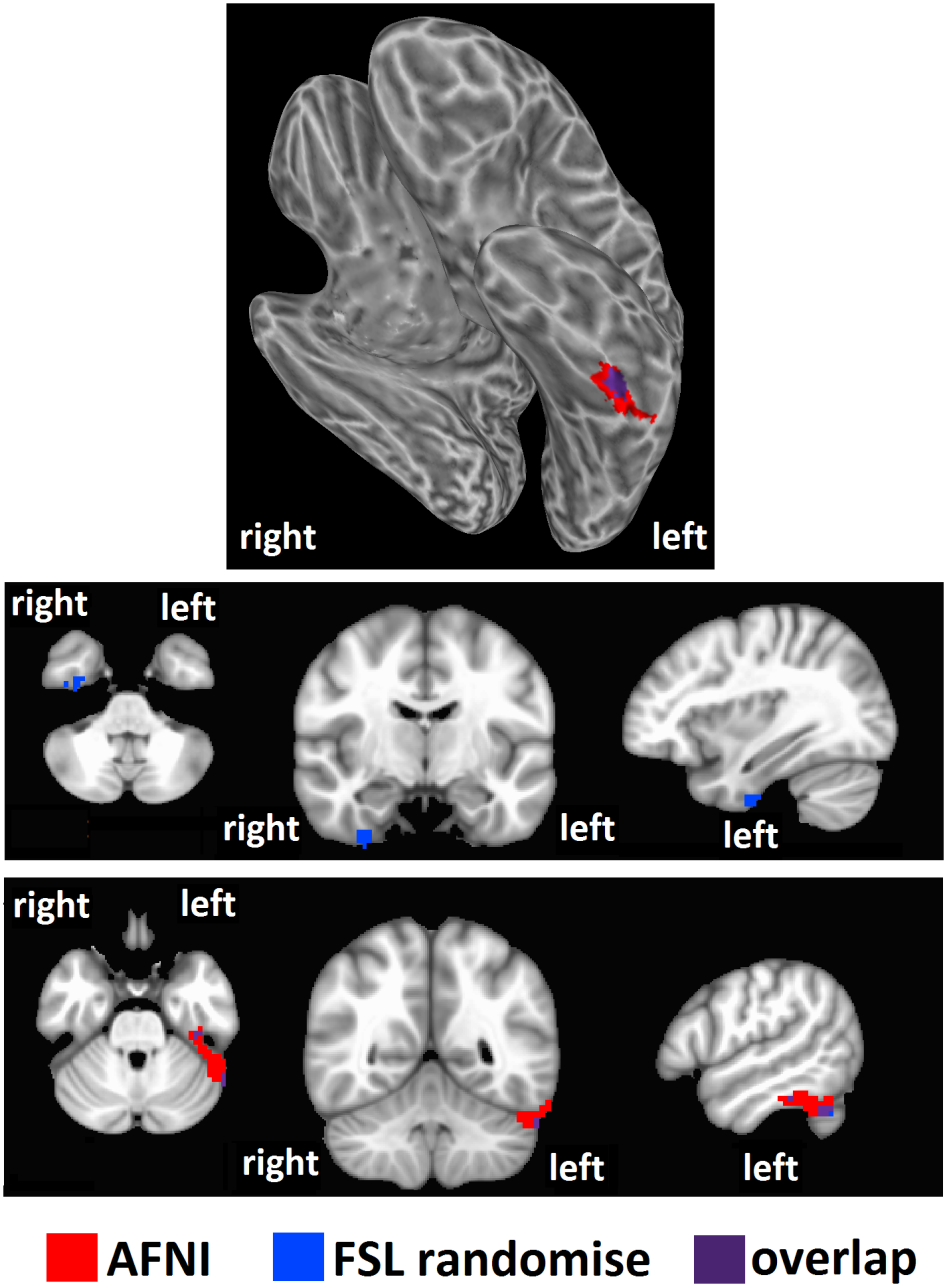
Brain entropy regression comparison with FSL randomise: Shipley estimated IQ. Whole brain regression analysis with brain entropy predicting Shipley estimated IQ was performed with SPM to compare to the AFNI results (*p* = 0.005). Results were in the positive direction, indicating increases in entropy predicting increases in IQ.

We tested our hypothesis that high intelligence would correspond to a high level of brain entropy in a sample of 892 healthy adults who participated in both resting-state fMRI and intelligence testing. Whole brain regression analysis, performed with AFNI, with intelligence estimated from Shipley vocabulary scores showed significant effects in which greater brain entropy predicted higher vocabulary scores (*t* = 2.811, corrected *p* = 0.001, Fig 1). This effect occurred in the left inferior temporal lobe, as well as the left superior cerebellum (see Table 1). Regression analysis performed by SPM found a similar, though smaller cluster (corrected *p* = 0.001, Fig 2). Regression analysis with FSL’s “randomise” yielded bilateral inferior temporal lobe clusters (*p* = 0.005, Fig 3), with the left cluster overlapping with the AFNI results. Results from these alternative methods were also in the positive direction, indicating that greater entropy predicted higher Shipley estimated IQ.

**Table 1:** Brain areas that showed a significant positive relationship between brain entropy and Shipley estimated IQ, as seen in Fig 1.

Region	Voxels	Peak	Center of Mass	Structures
Left inferior temporal lobe	187	-45, -39, -30	-50, -43, -26	Left fusiform gyrus Left inferior temporal gyrus Left parahippocampal gyrus Left cerebellum

Coordinates are in MNI space, LPI orientation.

**Fig 4:**
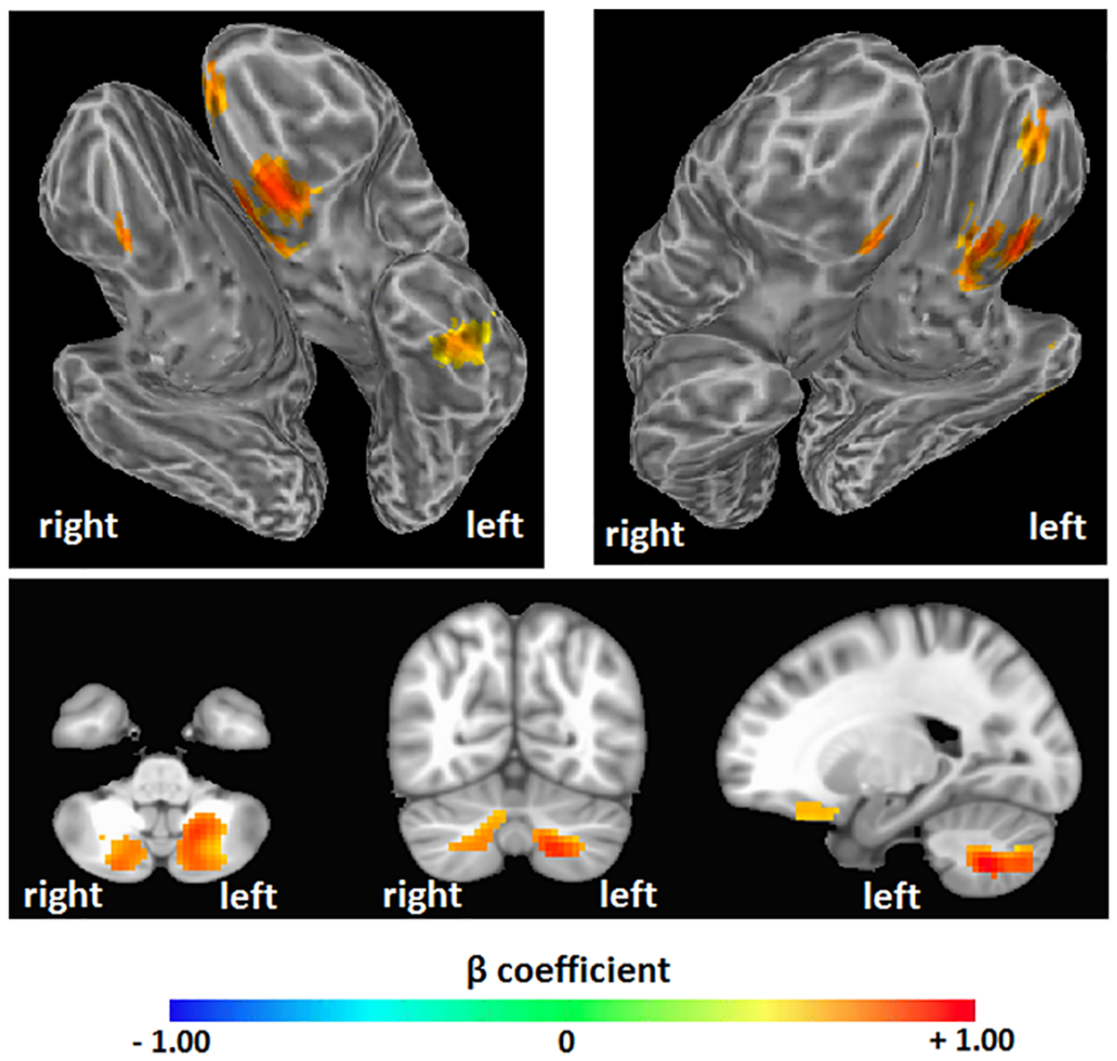
Brain entropy regression: WASI estimated IQ. Whole brain regression analysis was performed with AFNI to determine whether brain entropy predicts full-scale IQ as estimated from the Wechsler Abbreviated Scale of Intelligence, Matrix Reasoning subtest (uncorrected p = 0.005, cluster size = 157 voxels, corrected p = 0.001). Positive beta coefficients show where increases in resting-state brain entropy predict increases in IQ.

**Fig 5:**
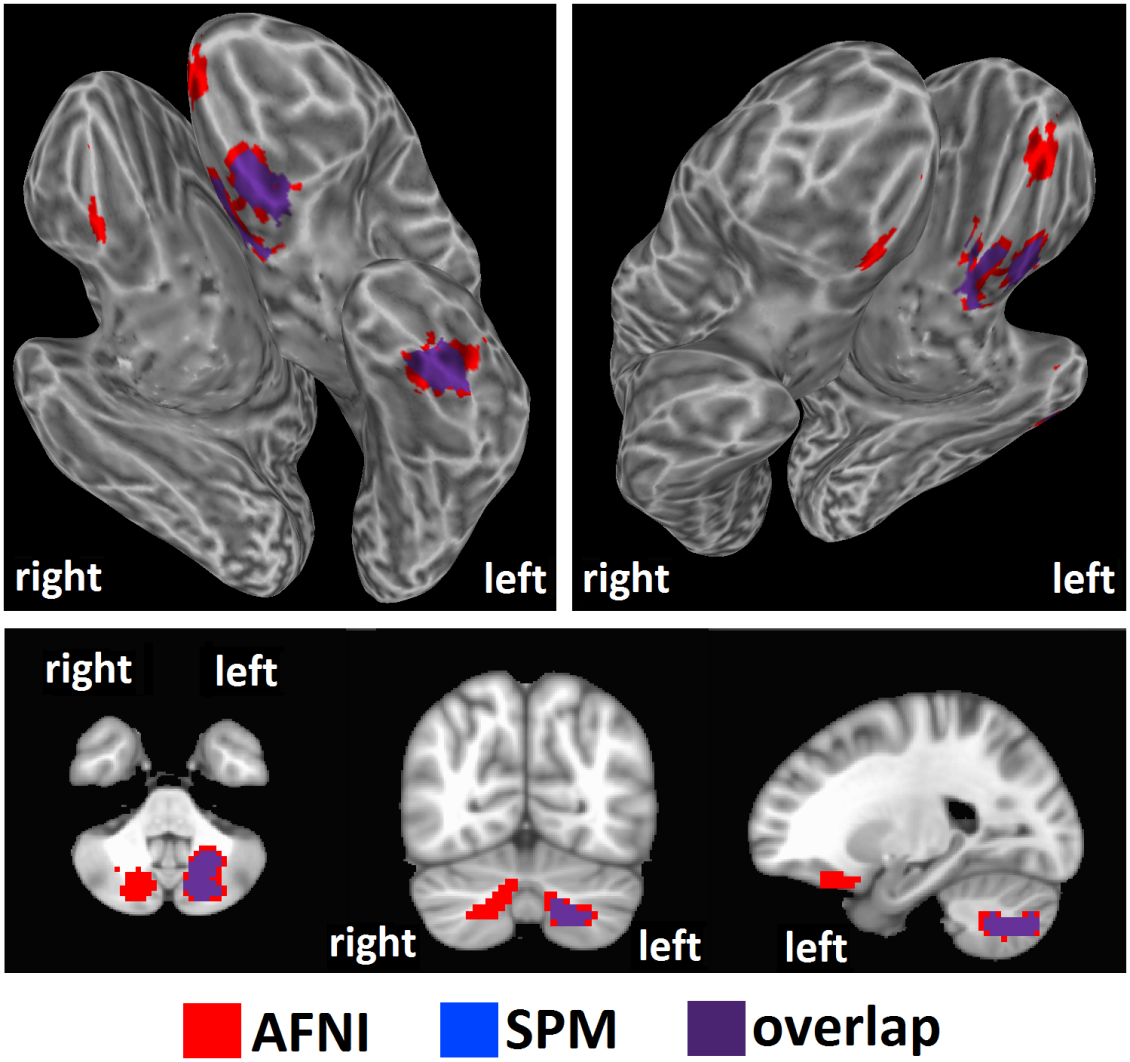
Brain entropy regression comparison with SPM: WASI estimated IQ. Whole brain regression analysis with brain entropy predicting WASI estimated IQ was performed with SPM to compare to the AFNI results (uncorrected *p* = 0.001, cluster size = 63 voxels, corrected *p* = 0.001). Results were in the positive direction, indicating increases in entropy predicting increases in IQ.

**Fig 6:**
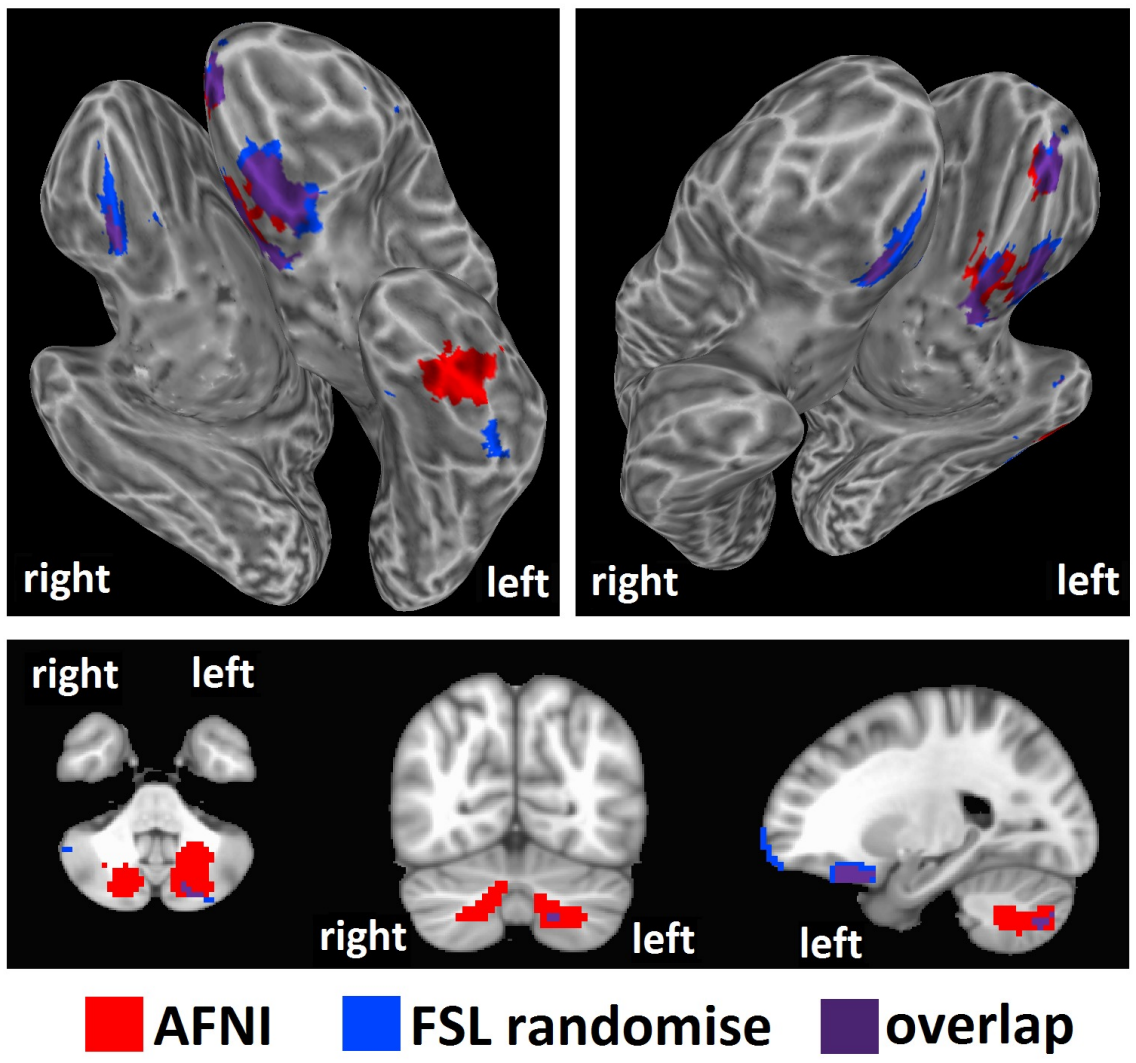
Brain entropy regression comparison with FSL randomise: WASI estimated IQ. Whole brain regression analysis with brain entropy predicting WASI estimated IQ was performed with SPM to compare to the AFNI results (*p* = 0.005). Results were in the positive direction, indicating increases in entropy predicting increases in IQ.

AFNI regression with intelligence estimated from WASI matrix reasoning scores (Fig 4) showed significant effects in bilateral anterior frontal and left inferior temporal areas, as well as bilateral cerebellum (see Table 2). Again, effects were in the positive direction, meaning that greater entropy predicted higher intelligence scores (*t* = 2.811, corrected *p* = 0.001, Fig 2). SPM regression analysis found similar clusters, though only in the left hemisphere (corrected *p* = 0.001, Fig 5). Regression with FSL’s “randomise” found largely overlapping clusters in the bilateral frontal areas, a slightly different and smaller cluster in the left inferior temporal region, and greatly reduced bilateral cerebellum clusters (*p* = 0.005, Fig 6). Results from these alternative methods were also in the positive direction, indicating that greater entropy predicted higher WASI estimated IQ.

**Table 2:** Brain areas that showed a significant positive relationship between brain entropy and WASI estimated IQ, as seen in Fig 2.

Region	Voxels	Peak	Center of Mass	Structures
Bilateral anterior frontal lobes	414	-9, 21, -15	-3, 29, -19	Bilateral superior orbital gyrus Bilateral rectal gyrus Bilateral middle frontal gyrus Bilateral medial frontal gyrus Bilateral inferior frontal gyrus Left subcallosal gyrus Left uncus
Left inferior temporal lobe	203	-54, -12, -33	-50, -14, -30	Left fusiform gyrus Left inferior temporal gyrus Left parahippocampal gyrus Left hippocampus
Left cerebellum	246	-18, -60, -45	-20, -67, -43	Left cerebellum
Right cerebellum	163	9, -78, -51	16, -73, -42	Right cerebellum

Coordinates are in MNI space, LPI orientation.

**Fig 7:**
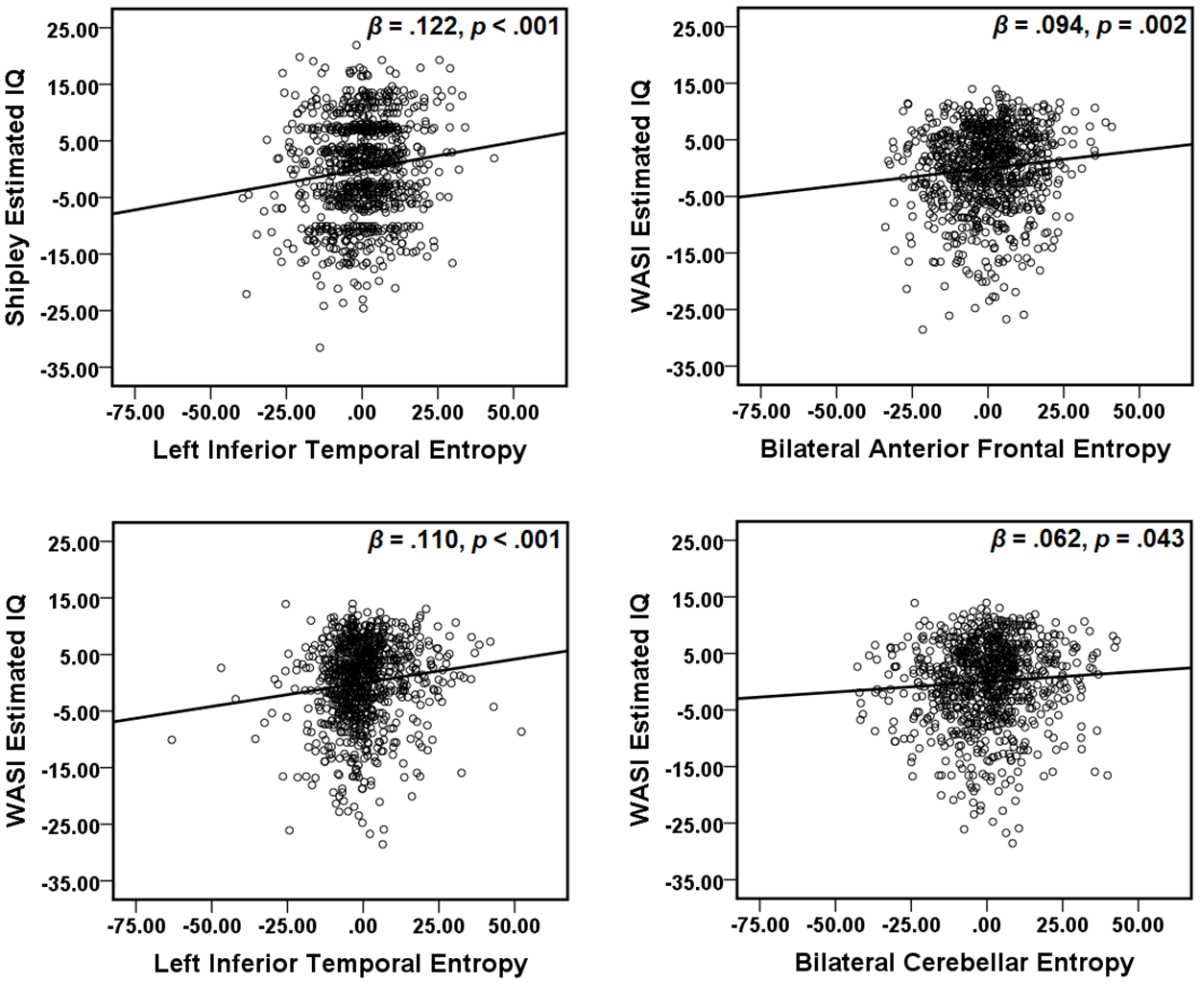
Partial regression plots. Multiple regressions were performed with both Shipley estimated IQ and WASI estimated IQ as dependent variables. Predictors included age, years of education, and average entropy values from fMRI ROIs associated with each IQ estimate. Plots show partial regression of brain entropy on estimated IQ after age and years of education were accounted for.

These areas showing a significant relationship between IQ estimates and entropy were defined as regions of interest [36]. The left and right cerebellum areas from the WASI estimated IQ analyses were combined into a bilateral cerebellum ROI. The average entropy value of each ROI was extracted from each participant. Multiple regression analyses were conducted with the two IQ estimates as dependent variables, and age, years of education, and average entropy from regions related to each IQ estimate as predictors (see Table 3). Age and years and education did not significantly predict Shipley estimated IQ, but brain entropy in the inferior temporal lobe did. Age and years of education significantly predicted WASI estimated IQ, but brain entropy in each ROI independently predicted IQ as well (Fig 7).

**Table 3:** *β* coefficients, *p*-values, and *R*^*2*^ values from multiple regression analyses with IQ estimates as dependent variables.

Dependent Variable	ROI	Entropy	Age	Years of Education
Shipleyestimated IQ	Left inferiortemporal lobe Overall *R*^*2*^ = 0.018	*β* = 0.122 *p* < 0.001 *R*^*2*^ = 0.015	*β* = -0.052 *p* = 0.376 *R*^*2*^ < 0.001	*β* = 0.086 *p* = 0.144 *R*^*2*^ = 0.002
WASI estimated IQ	Bilateral anteriorfrontal lobes Overall *R*^*2*^ = 0.198	*β* = 0.094 *p* = 0.002 *R*^*2*^ = 0.011	*β* = 0.171 *p* = 0.001 *R*^*2*^ = 0.012	*β* = 0.274 *p* < 0.001 *R*^*2*^ = 0.029
	Left inferiortemporal lobe Overall *R*^*2*^ = 0.202	*β* = 0.110 *p* < 0.001 *R*^*2*^ = 0.015	*β* = 0.168 *p* = 0.002 *R*^*2*^ = 0.011	*β* = 0.281 *p* < 0.001 *R*^*2*^ = 0.031
	Bilateral cerebellum Overall *R*^*2*^ = 0.193	*β* = 0.062 *p* = 0.043 *R*^*2*^ = 0.005	*β* = 0.162 *p* = 0.002 *R*^*2*^ = 0.010	*β* = 0.282 *p* < 0.001 *R*^*2*^ = 0.031

**Fig 8:**
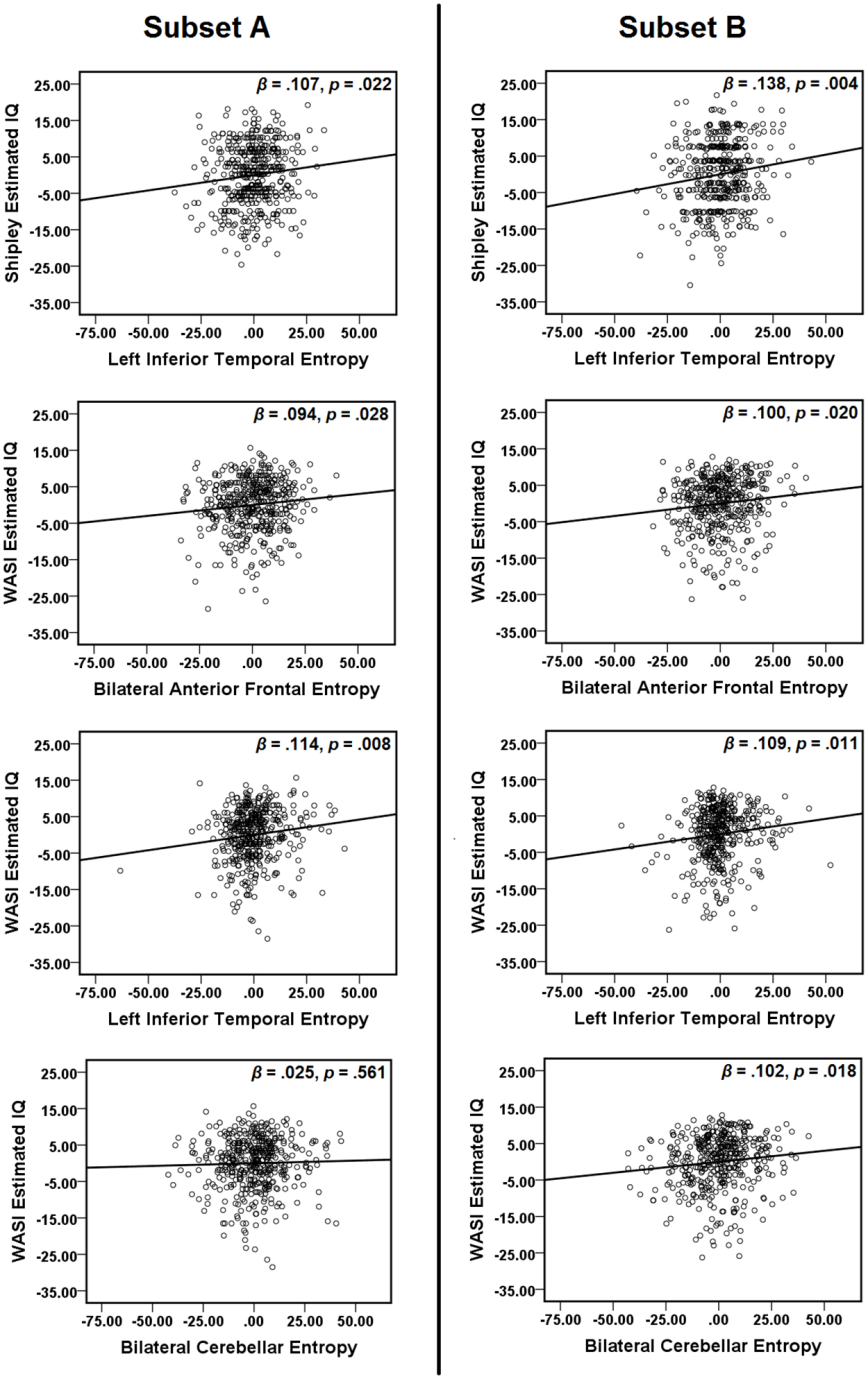
Partial regression plots for random subsets. The data set was split randomly into two subsets of approximately 50%. For each subset, multiple regressions were performed with both Shipley estimated IQ and WASI estimated IQ as dependent variables. Predictors included age, years of education, and average entropy values from fMRI ROIs associated with each IQ estimate. Plots show partial regression of brain entropy on estimated IQ after age and years of education were accounted for.

Reliability was assessed by splitting the data into two randomly selected subsets of approximately 50% of the total data (n = 454 for subset A, n = 438 for subset B), and performing multiple regressions for each subset (Fig 8). The subsets did not significantly differ in age (*t* = 1.131, *p* = 0.258; subset A: 21.51 ± 2.82, subset B: 21.72 ± 2.86, mean ± standard deviation), years of education (*t* = 0.731, *p* = 0.465; subset A: 14.54 ± 1.93, subset B: 14.64 ± 1.96), Shipley estimated IQ (*t* = -0.980, *p* = 0.327; subset A: 114.04 ± 8.54, subset B: 113.46 ± 9.09), or WASI estimated IQ (*t* = 1.314, *p* = 0.189; subset A: 108.37 ± 7.97, subset B: 109.09 ± 8.33). For both subsets, brain entropy predicted Shipley estimated IQ as it did in the overall data set (see Table 4). In subset A, Shipley IQ was additionally predicted by years and education, and at a trend level, by age. For WASI estimated IQ, the results in subset B matched the pattern of results for the overall data set. However, subset A deviated from these results in the following ways: brain entropy failed to predict IQ in the bilateral cerebellum ROI, and age failed to predict IQ in all ROIs.

**Table 4:** Results from two randomly selected subsets, A and B, of approximately 50% of the overall data set: *β* coefficients, *p*-values, and *R*^*2*^ values from multiple regression analyses with IQ estimates as dependent variables.

Dependent Variable	ROI	Subset	Entropy	Age	Years of Education
Shipley estimated IQ	Left inferior temporal lobe	A Overall *R*^*2*^ = 0.026	*β* = 0.107 *p* = 0.022 *R*^*2*^ = 0.012	*β* = -0.150 *p* = 0.064 *R*^*2*^ = 0.008	*β* = 0.201 *p* = 0.013 *R*^*2*^ = 0.013
		B Overall *R*^*2*^ = 0.020	*β* = 0.138 *p* = 0.004 *R*^*2*^ = 0.019	*β* = 0.053 *p* = 0.534 *R*^*2*^ < 0.001	*β* = -0.033 *p* = 0.697 *R*^*2*^ < 0.001
WASI estimated IQ	Bilateral anterior frontal lobes	A Overall *R*^*2*^ = 0.190	*β* = 0.094 *p* = 0.028 *R*^*2*^ = 0.011	*β* = 0.078 *p* = 0.292 *R*^*2*^ = 0.002	*β* = 0.351 *p* < 0.001 *R*^*2*^ = 0.048
		B Overall *R*^*2*^ = 0.210	*β* = 0.100 *p* = 0.020 *R*^*2*^ = 0.012	*β* = 0.266 *p* = 0.001 *R*^*2*^ = 0.027	*β* = 0.193 *p* = 0.013 *R*^*2*^ = 0.014
	Left inferior temporal lobe	A Overall *R*^*2*^ = 0.194	*β* = 0.114 *p* = 0.008 *R*^*2*^ = 0.016	*β* = 0.084 *p* = 0.255 *R*^*2*^ = 0.003	*β* = 0.348 *p* < 0.001 *R*^*2*^ = 0.048
		B Overall *R*^*2*^ = 0.212	*β* = 0.109 *p* = 0.011 *R*^*2*^ = 0.015	*β* = 0.251 *p* = 0.001 *R*^*2*^ = 0.025	*β* = 0.211 *p* = 0.006 *R*^*2*^ = 0.017
	Bilateral cerebellum	A Overall *R*^*2*^ = 0.182	*β* = 0.025 *p* = 0.561 *R*^*2*^ < 0.001	*β* = 0.082 *p* = 0.273 *R*^*2*^ = 0.003	*β* = 0.352 *p* < 0.001 *R*^*2*^ = 0.048
		B Overall *R*^*2*^ = 0.211	*β* = 0.102 *p* = 0.018 *R*^*2*^ = 0.013	*β* = 0.205 *p* = 0.008 *R*^*2*^ = 0.024	*β* = 0.250 *p* = 0.001 *R*^*2*^ = 0.016

The *β* coefficients showing the relationship between entropy and estimated IQ were modest, and often smaller than the *β* coefficients for age and years of education. While age and education are pervasive variables that affect all aspects of functioning, brain entropy was here measured in a resting-state fMRI scan, which represents a specific mode of brain activity. The presence of significant relationships between entropy and estimated IQ indicates that general access to a variety of brain states predicts performance in these two specific tasks. The modesty of the relationships indicates that this general entropy may comprise a readiness to engage in a large variety of tasks beyond these specific IQ measures. Stronger relationships might be obtained using brain entropy from active task performance, where brain activity is focused on the specific mode of thinking required for the task.

**Fig 9:**
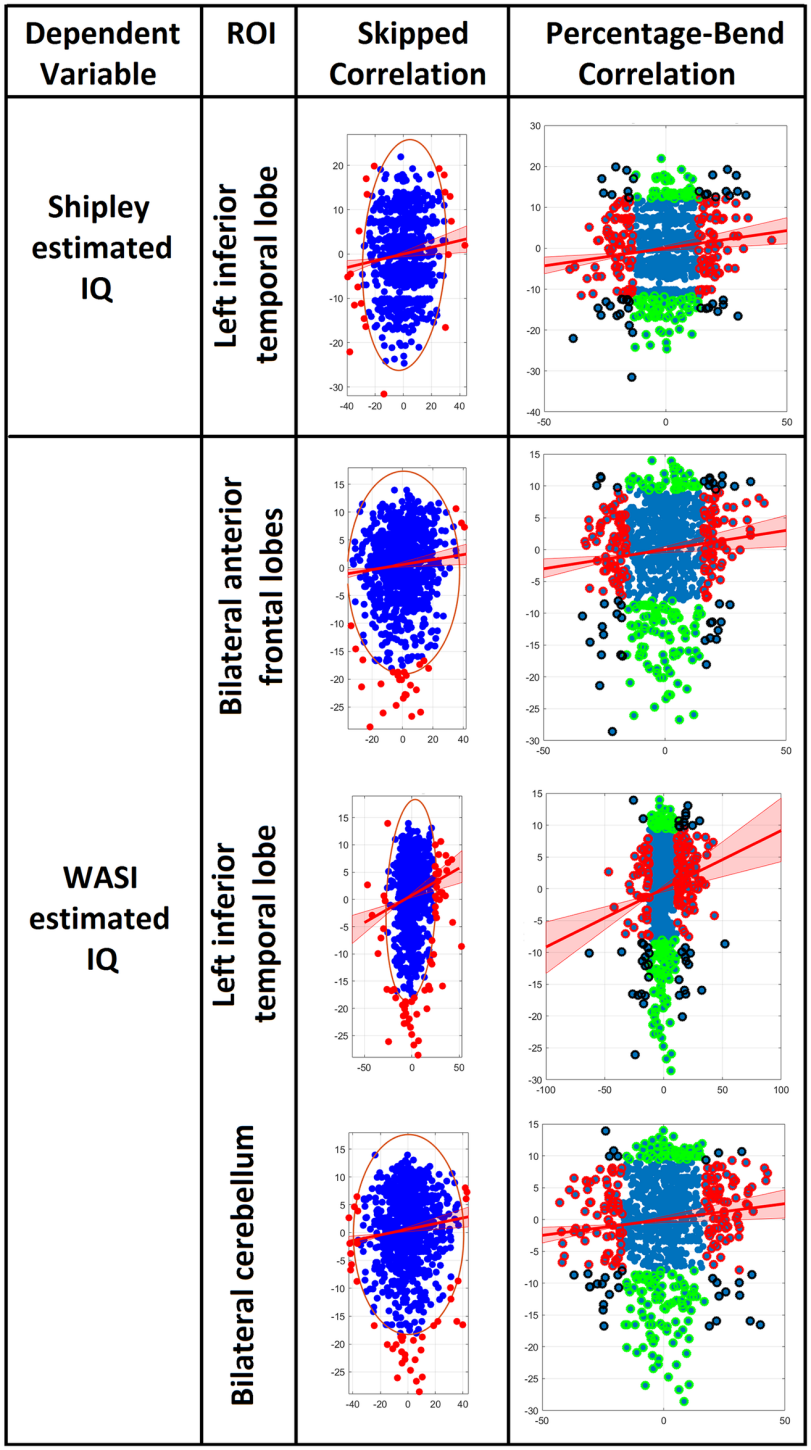
Partial regression plots using skipped correlation and percentage-bend correlation. Plots show partial regression of brain entropy on estimated IQ after age and years of education were accounted for. All X and Y axes are residuals for partial regression. For skipped correlations, ellipses contain non-outlying data, red lines represent best linear fit, and red data points are bivariate outliers removed based on normality. For percentage-bend correlations, red and green data points are outliers for X and Y respectively, and black data points are outliers for both X and Y. Red, green, and black data points were down-weighted. Analyses were performed with the Robust Correlation Toolbox for MATLAB [37].

Due to the modest size of these partial regression relationships between entropy and estimated IQ, we further tested them using the Robust Correlation Toolbox for MATLAB [37], to confirm their significance. The authors of this program showed that when data were not normally distributed, the Pearson correlation could produce serious errors due to the influence of outliers. The Henze-Zirkler test for multivariate normality [38] showed that our data were not normally distributed, except for the relationship between anterior frontal entropy and WASI estimated IQ (Table 5). We therefore computed the partial regression correlations predicting estimated IQ with entropy by using the Pearson and Spearman skipped-correlation [39–41], which ignores outliers by using the overall structure of the data, and the percentage-bend correlation, which down weights 20% of marginal observations (Table 5, Fig 9). These methods yielded significant results for all partial regression correlations, confirming that these modest relationships are robust.

**Table 5:** Robust analysis for partial regression correlations with regional entropy predicting estimated IQ: Henze-Zirkler test for multivariate normality (HZ, significant result indicates non-normality), Pearson skipped correlation (significance determined for alpha = 5%), and percentage-bend correlation.

Dependent Variable	Entropy ROI	Normality	Skipped Correlation	Percentage-Bend Correlation
Shipley estimated IQ	Left inferior temporal lobe	HZ = 3.289 *p* < 0.001	Pearson *r* = 0.090 Spearman *r* = 0.095 both significant	*r* = 0.110 *p* = 0.001
WASI estimated IQ	Bilateral anterior frontal lobes	HZ = 0.674 *p* = 0.241	Pearson *r* = 0.083 Spearman *r* = 0.094 both significant	*r* = 0.101 *p* = 0.003
	Left inferior temporal lobe	HZ = 23.081 *p* < 0.001	Pearson *r* = 0.129 Spearman *r* = 0.131 both significant	*r* = 0.134 *p* < 0.001
	Bilateral cerebellum	HZ = 5.434 *p* < 0.001	Pearson *r* = 0.099 Spearman *r* = 0.100 both significant	*r* = 0.093 *p* = 0.006

## Discussion

The results confirmed our hypothesis that greater brain entropy is associated with higher intelligence. All effects found by whole-brain regression analyses performed by three separate methods were in the positive direction. Subjects with higher verbal and performance intelligence estimates, measured with Shipley vocabulary and WASI matrix reasoning scores, respectively, had higher brain entropy. In particular, estimates based on vocabulary were found to be most strongly related to brain entropy in the left inferior temporal lobe. This region is known to be involved in reading [42], word retrieval [43], and semantic retrieval [22–25]. Part of the left superior cerebellum also showed this relationship between entropy and intelligence, and recent work suggests important roles for the cerebellum in phonological processing and storage, verbal retrieval, and reading [44]. Variability of brain signals in these particular areas may thus relate to the ability to readily retrieve words and their meanings, and/or a larger repertoire of stored vocabulary. Estimates based on matrix reasoning scores were also found to be related to brain entropy in the left inferior temporal lobe. Additionally, these estimates related to entropy in bilateral prefrontal areas, which are important for fluid intelligence involved in solving novel problems more so than for crystalized intelligence involved in accessing stored knowledge such as vocabulary [26, 27]. The involvement of both temporal and frontal entropy may indicate the importance of both crystalized and fluid intelligence in complex reasoning tasks. Intelligence estimates from matrix reasoning were also predicted by entropy in the cerebellum, which contributes to spatial representation [45] and is implicated in visual-spatial, and executive abilities [46], which are critical for the Matrix Reasoning task.

This pattern of results was derived from brain entropy measured during the resting state. According to our hypothesis, entropy in this context provides an indicator of the brain’s general readiness to process unpredictable stimuli from the environment, rather than the active use of brain states during a particular task. It is likely that brain entropy during a task, such as the Vocabulary or Matrix Reasoning tasks utilized here, would be more strongly linked to performance. However, our results confirm that general resting-state entropy does predict performance on specific intelligence measures, and that it does so differently for Vocabulary and Matrix Reasoning measures. Consistent with the idea that resting-state entropy represents general readiness for unpredictable stimuli, the Matrix Reasoning task, which requires reasoning in flexible ways about novel stimuli, was linked to entropy in a variety of widespread regions. The Vocabulary task, which relies on stored knowledge, showed fewer links to resting-state entropy.

Multiple regression analyses showed that although the age and educational experience of participants also predicted intelligence, the relationships between regional resting-state brain entropy and intelligence were independent of these variables. Further, the ability of brain entropy to predict intelligence held true for two randomly selected subsets of the data, with the only exception of cerebellar entropy in one of the subsets. Together, these results suggest that entropy is a reliable predictor of intelligence, and provides unique information not captured by developmental and educational status alone.

How do these results illuminate the nature of human intelligence? Our findings are consistent with—and may provide empirical support for—an influential theory on the nature of brain functioning related to the minimization of free-energy. Friston and colleagues provide an entropic account of adaptive brain function based on Bayesian principles [13, 47, 48]. According to this perspective, a central function of the brain is to minimize free-energy. Free-energy minimization is the motivation of intelligent systems to avoid surprising states (e.g. a fish avoids the experience of a water-less state). The brain minimizes such surprising states with an established model-of-the-world and samples sensory information to infer the causes of processed sensation. With this model-of-the-world, the brain uses Bayesian probability to make predictions of understood causal processes to avoid surprises. The brain’s model-of-the-world is represented via established recognition densities to be used to process sensation (and inferred causes). An unpredicted state-of-the-world would result from such a state not being properly represented in the brain’s recognition density (and would constitute a surprise). Importantly, a brain with a recognition density that can correlate with a very high number of possible environmental/causal events would be much less likely to find itself surprised than would a brain with a recognition density that can correlate with fewer possible states of the world. The number of possible brain states that can be deployed within a recognition density would define a brain’s entropy. Thus, the Free-Energy theory of the brain would also associate intelligence with high brain entropy. Importantly, the Free-Energy theory of the brain integrates the notion of *active inference* as a central means by which the brain avoids surprising states [49]. Active inference is a fundamental process for making the brain’s prior recognition probability distribution, a posterior probability distribution (or minimize the Kullback-Leibler divergence between the hidden posterior and the prior distributions). Briefly, *active inference* involves inference on hidden states of the world and future control states given prior beliefs about action. Accordingly, a central inferential task of the brain involves minimizing the “…divergence between prior goals—over final states—and conditional distributions, given the future state of the world and future choices” [49]. Active inference is adaptive by maximizing entropy over outcomes such that information gain, exploration, and ‘keeping of options open’, is prioritized. Such a tendency to maximize entropy via active inference would describe a brain poised to make accurate predictions about a large number of possible outcomes. Integrating these ideas, the high brain entropy levels of subjects in this study may reflect a state where the Kullback-Leibler divergence has already been minimized, compared to subjects with less entropic brains. Our findings, with a large sample of healthy adult subjects, may serve to provide empirical support for this theoretical literature on the specific role of entropy in the brain.

There are several promising avenues for future research building from our findings. Although we sought to test our hypotheses using resting-state fMRI, there would be great value in examining brain entropy change between the resting-state and specific cognitive tasks. A recent functional connectivity study found that higher intelligence was associated with smaller changes between resting-state functional networks and task-related networks [50]. This suggests intelligence is related to efficiency in adapting to task demands, which is supported by our finding that higher intelligence relates to more flexible access to brain states at rest.

### Limitations

While our findings support our specific hypothesis, our conclusions for the broader theory of brain entropy are limited in ways that future work should address. The participants had a higher average IQ compared to the general population, and it is unknown to what extent the relationship between intelligence and entropy extends to other IQ ranges, particularly extreme ranges. Studies relating entropy to intellectual disability as well as highly elevated intelligence would further elucidate the specific role of entropy in intellectual functioning. The data set also used IQ estimates, rather than full IQ batteries. Future studies with more detailed IQ testing could reveal the involvement of entropy in specific brain regions with various aspects of intelligence. The use of fMRI signals to calculate entropy over time also contains inherent limitations. The time scale of fMRI is on the order of seconds, whereas methods such as electroencephalography (EEG) can reveal changes in brain activity on the millisecond scale. As brain entropy is a measure of variability in activity over time, examining variability at finer time scales may reveal properties of entropy not detectable by fMRI. Finally, our study utilized resting-state fMRI data. Future studies should investigate the role of entropy during active task performance, which may yield larger effect sizes for relationships between entropy and task performance.

## Conclusion

This study used fMRI to examine brain entropy in a large sample of healthy adults and found that higher entropy in several key brain areas predicted higher intelligence measures, in both verbal and performance measures. Our findings fit with current theories of how brain systems may function through entropic principles. Further work is needed to explore the precise nature of this relationship between entropy and intelligence, as well as to elucidate it in populations with intellectual or other brain processing impairment. The link between functional diversity of brain states and intellectual ability suggests that intelligence may be assisted by therapies or other treatments that increase availability of and access to varied neurological states.
